# Characterizing pen strokes produced using various commercially available thermochromic inks

**DOI:** 10.1093/fsr/owae055

**Published:** 2025-01-12

**Authors:** Mario Alejandro Alvarez Cordeiro, Catalina Gondikas

**Affiliations:** Córdoba, Argentina; Córdoba, Argentina

**Keywords:** forensic sciences, thermochromic, thermosensitive, erasable, pen, ink

## Abstract

The characteristics of commercially available thermochromic ink pens have been studied and described since their appearance in 2006. The wide variety of brands and models now available warrants further study using an expanded sample size, to differentiate the general characteristics from specific characteristics. Herein, the ink strokes of 15 pens purchased in the province of Córdoba, Argentina were studied. First, the initial unaltered strokes were examined. Second, heat-manipulated strokes (with and without friction) were evaluated. Several characterization techniques were employed, such as observation by the naked eye, optical magnification, and light irradiation using different spectral bands. In 100% of the unaltered strokes, the general characteristics of thermochromic inks, such as ink accumulation at the end of the stroke path and a “pasty appearance”, were found. It was possible to visualize colourless strokes (i.e. erased strokes) based on their contrast with the paper by shining oblique light at an opposite angle to that of the observation. In addition, the responses to ultraviolet (UV) (365 and 254 nm) and cyan light (505 nm) by infrared techniques were useful for distinguishing the thermochromic ink. Specific characteristics, such as high relief in areas of ink accumulation, less ink accumulation in the internal zone of the stroke, and intensity differences in infrared radiation (IR) luminescence emitted by different brands and models, could be useful in determining the writing tool that was used.

**Key points:**

- Thermochromic ink disappears with heat but is reversible with cold.
- UV techniques and IR photography serve to make thermochromic ink visible.
- Initially use oblique white light opposite to the angle of observation to make strokes visible.

## Introduction

This study aimed to determine the characteristics that can be used by the forensic document examiner to identify the existence of a pen stroke produced using a thermochromic ink pen. Although several reports refer to these characteristics [1–4], the limited sampling in those studies and the recent appearance of numerous models and brands on the market necessitate a new study to verify the characteristics present in the strokes produced for this type of pen. Therefore, we evaluate 15 writing tools sold in bookstores in the province of Córdoba, Argentina and provide support to the forensic examiner when approaching the analysis of a document where this ink may be present in different states, namely visible and not visible. This analysis reveals the behaviour of the inks when subjected to different spectral bands, thereby defining the general characteristics that can be used to determine whether the pen strokes were produced using this type of writing tool, and in turn, elucidating whether there are specific characteristics that can be used to identify the type of thermochromic ink pen (colour, brand, and model).

Before starting this study, it is necessary to consider the historical context and explain the functionality of thermochromic ink pens. The historical information is relevant in establishing the approximate date on which a stroke could have been produced using this type of pen. For this, the reference date is determined using one of the first thermochromic ink pen models created by the Japanese company Pilot [5]. It was launched in the Asian market in 2007, the year after its launch in Europe [6]. Notably, the ink becomes colourless (i.e. not visible) when subjected to temperatures of 65°C, a phenomenon that is generated by friction using the special plastic rubber located at the upper end of the barrel of the tool (i.e. the eraser). To make the ink visible again, it must be subjected to a temperature of ~−20°C. This reaction occurs because the ink is composed of microcapsules that form the pigmentation and contain an equal mixture of three substances: a leuco dye that can change between coloured and colourless states, a colour developer, and a colour change temperature regulator. The leuco dye determines the colour but does not produce it until it chemically bonds with the developer. The colour change temperature regulator prevents bonding above a certain temperature, making the colour disappear [6]. Although Pilot warns on its official website that this instrument should not be used to make signatures or fill out legal documents, and although it had a positive impact on primary education, we have to consider the possibility that someone may use it to commit fraud. Therefore, with the emergence of new thermochromic writing instruments from other brands, often at lower price points, the possibility of finding documents altered with the help of this type of pen grows, as well as the need to learn more about the characteristics of the strokes produced using this ink.

In 2008, 2 years after the launch of Pilot FriXion, Carmen Vercher, a forensic document examiner from the Superior Court of Justice in Galicia, Spain, was in charge of analysing the characteristics and features of this new pen. The text erased by friction leaves a slight anomaly on the paper, providing several clues. The text that has been removed by heating can go unnoticed and may be confused with an indentation, but with the appropriate equipment, it can be recognized. The erased inscriptions were revealed by IR photography under the luminescence mode and UV irradiation using 313 nm light [4].

Subsequently, other characteristics of the strokes made with the new pen created by the Pilot company were described. The stroke produced by this tool can be recognized by studying it under a low-magnification microscope. The appearance is like that of the surface of a “caoutchouc”, which has a “pasty” aspect. Likewise, some strokes are observed with a lower ink load. After erasing by friction and applying a raking light, the indentation left when writing can be revealed. Likewise, the ink also disappears when subjected to heat; if the written paper is subjected to temperatures of freezing, the line reappears, although less inked than before being erased [4]. Furthermore, the report describes the reduced ink accumulation in the internal zone of the pen stroke.

Other studies conducted in Egypt also highlight the high IR fluorescence of these inks [1, 2].

A 2016 study on two Pilot FriXion pens confirmed the use of VSC-6000/HS to detect fraud performed using this writing instrument [7].

Using three colours of Pilot FriXion ink, another study concluded that “...FriXion ink pen strokes of all colours could be distinguished successfully under ultraviolet lighting. Under infrared luminescence, faded and fresh blue FriXion ink could be differentiated at most preset excitation wavelengths of infrared luminescence…”[8].

In 2017, another study was conducted using a ballpoint pen with blue thermosensitive gel ink and a hard rubber eraser, namely Genio from the Simball brand, aiming to determine whether there were differences between writing that was erased minutes after it was made and writing that was erased 47 days after it was made [3]. The study applied and analysed two erasing methods: heat by friction, using the rubber incorporated in the cap of the pen, and heat by convection, using a manual hair dryer. Furthermore, the study sought to observe the differences in writings that were created and erased at different times, after they reappeared by subjecting them to a temperature of −14°C for 2 min. Finally, they compared the erased samples under light of different wavelengths and angulation to determine the characteristics of the strokes. Ultimately, a greater interval between the production of the writing and its erasure led to more visible signs. Furthermore, the best erasing results were obtained by friction and not by convection because the same intensity of heat was more homogeneous using the hard rubber. However, friction also affects the physical characteristics of the indentations, regardless of the time elapsed. Moreover, it was shown that the erased writings can be read by exploiting the difference in contrast, both with raking visible light (owing to the difference in relief) and with UV radiation and IR luminescence techniques.

In 2021, a study on Pilot FriXion pens (blue colour) concluded that, “In all other cases restoration by putting the samples in a refrigerator is possible” [9].

Although this previously published work provides important information of forensic interest, the list of potential characteristics remains unclear, particularly because of the few samples that have been submitted for analysis. It is necessary to determine the various characteristics of the inscriptions made with thermochromic ink pens of other brands and models.

This study aims to characterize the strokes inscribed using different commercial thermochromic ink pens sold in the province of Córdoba, Argentina. Photographic techniques are employed using visible and non-visible spectral bands, discriminating the general characteristics that are present in 100% of the samples from the specific characteristics observed in only a few samples.

## Methodology

### Materials

**Figure 1:**
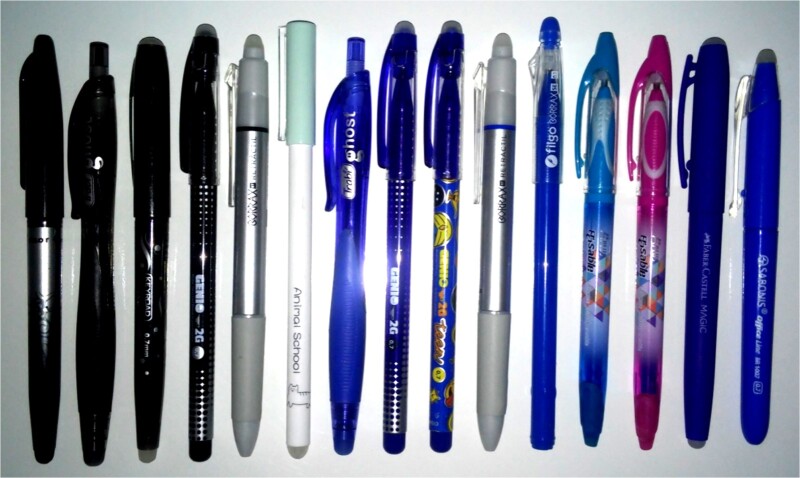
The thermochromic ink pens used in this study. From left to right. Black colour: Pilot FriXion, Trabi Ghost, Keyroad Erasable Gel Pen, Simball Genio 2G 0.7, Filgo Borrax RT Retractable, and Zuixua Animal School K 1300 0.5 mm. Blue colour: Trabi Ghost, Simball Genio 2G 0.7, Simball Genio Teen 2G 0.7, Filgo Borrax RT Retractable, Filgo Borrax SE 0.7, A+ Plus Erasable GA304205E 0.7 (tube: light blue), A+ Plus Erasable GA304205E 0.7 (tube: pink), Faber Castell Magic, and Sabonis Office Line BR1007 0.7.

- Black thermochromic ink pens: Pilot FriXion, Trabi Ghost, Keyroad Erasable Gel Pen, Simball Genio 2G 0.7, Filgo Borrax RT Retractable, and Zuixua Animal School K 1300 0.5 mm (Figure 1).

-Blue thermochromic ink pens: Trabi Ghost, Simball Genius 2G 0.7, Simball Genio Teen 2G 0.7, Filgo Borrax RT Retractable, Filgo Borrax SE 0.7, A+ Plus Erasable GA304205E 0.7 (tube: light blue), A+ Plus Erasable GA304205E 0.7 (tube: pink), Faber Castell Magic, and Sabonis Office Line BR1007 0.7 (Figure 1).

-A4 sheets of commercial paper, matte, 75 g/m^2^.

-Graphite pencil.

-Lighter.

-Freezer (−18°C).

### Instrumental

*Optical*

- Arcano stereoscopic magnifying glass (20 and 40×).

- Nikon D5100 camera.

- LG-D722AR camera.

- Nikon D3400 camera (full spectrum).

- Neewer high-pass IR filter (720 nm).

*Lighting*

- LED ZY-5208B flashlight (white light).

- Spectroline MiniMAX portable UV lamp (365 and 254 nm).

- Biolight LED forensic lights (505 and 850 nm).

Two comparative tables (Tables A and B) are prepared on separate sheets of paper, as follows:

The first column is headed with the title “Writing Instrument”, listing the names of the writing tools, placing the black pens first, followed by the blue ones. A graphite pencil is used for this list. In each row (for each sample), the word “thermochromatic” is written repeatedly and uninterruptedly until reaching the end of the page, using the corresponding pen named in the first column.

**Figure 2:**
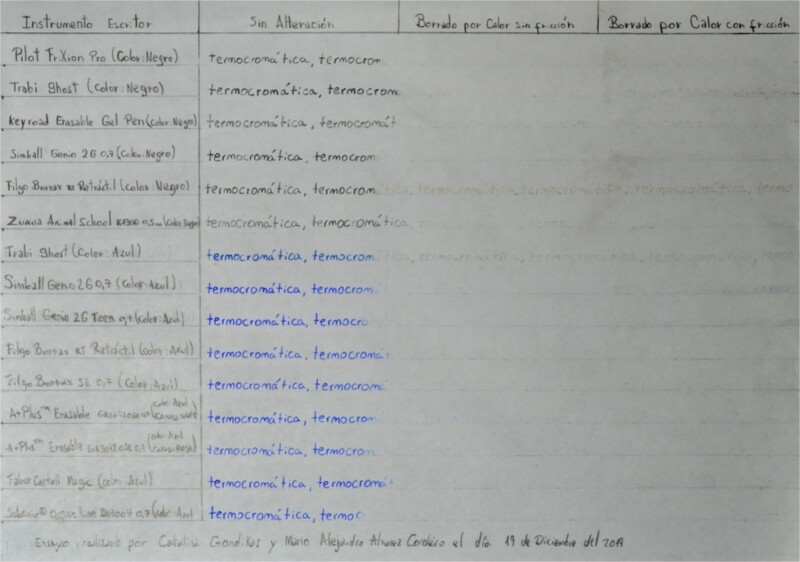
Table A, incident white light. Photography: Nikon D5100 camera; exposure time: 1/2 s; F: 9; ISO: 100.

For Table A, we have three additional columns within the area where the writing is performed using the thermochromic pens. Within the first additional column, the text is maintained without any alteration. In the second additional column, the text is erased by heat without friction, with the help of a lighter, and in the third additional column, the text is erased by heat with friction using the plastic eraser attached to each tool (Figure 2). The ink is immediately erased once the filling of the writing samples is completed.

**Figure 3:**
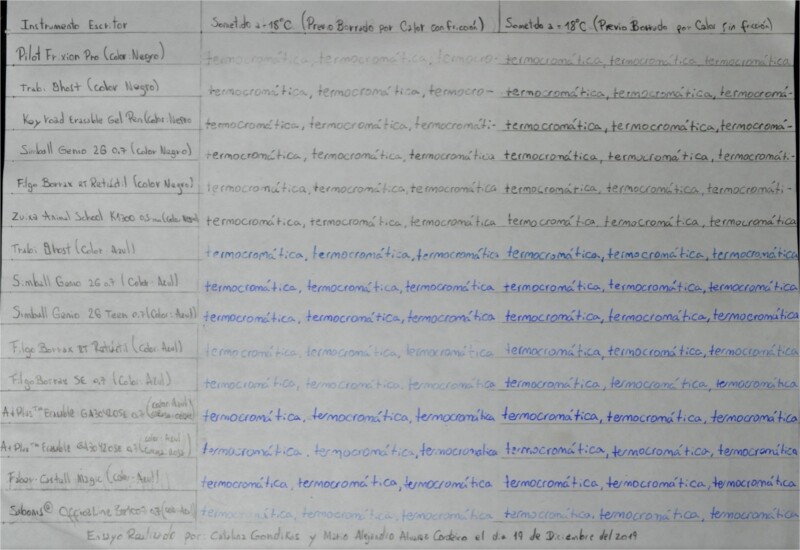
Table B, incident white light. Photography: Nikon D5100 camera; exposure time: 1/4 s; F: 9; ISO: 100.

Table B proceeds in the same way as Table A, except the writing sample area is divided into two additional columns within which the text is manipulated using the two heating methods used in the previous table, and then the document is placed in a freezer for 10 min (Figure 3). Again, the ink is immediately erased once the filling of the writing samples is completed.

The analysis of the writing includes five possible use cases:

- Unaltered.

- Colourless (heat and friction).

- Colourless (heat without friction).

- Tempered at −18°C (after manipulation by heat without friction).

- Tempered at −18°C (after manipulation by heat with friction).

### Techniques applied for the first three variables obtained (Table A)

 - Naked eye observation.

- Observation using a stereoscopic magnifying glass.

- UV radiation at 365 and 254 nm.

- IR digital photography, reflection mode.

- IR digital photography, luminescence mode.

In the case of reappeared text, Table B, only the possibility of recovering the visibility of the ink is evaluated. In all cases, outstanding characteristics in the literature review and new findings that may result from this analysis are considered.

#### Naked eye observation

The evaluation begins by observing the unaltered texts without instruments, to see if they present clear characteristics that can be used to identify the presence of thermochromic ink.

After this, the strokes are evaluated in a colourless state from a zenith viewing angle (i.e. perpendicular to the document), and then the colourless strokes are observed while varying both the angle of observation and the incidence of light. The purpose of this technique is to determine how “invisible” the texts are after being manipulated by heat and determine whether they leave visible residues that allow the readability of the text through careful observation.

Using this technique, we also determine whether the colour reappears at −18°C.

#### Observation using a stereoscopic magnifying glass

Using a stereoscopic magnifying glass and a white LED flashlight, the presence of unequivocal signs of identification is evaluated in a natural and colourless state.

Uncommon characteristics within the sample group are also relevant because they can help to identify or rule out specific writing tools (brand and model).

#### UV radiation at 365 and 254 nm

A portable Spectroline MiniMAX lamp is used to emit UV radiation with wavelengths of 365 and 254 nm, the possibility of recognizing the original text after erasure is examined.

#### IR digital photography in reflection mode

A Nikon D3400 full spectrum camera with a 720 nm highpass IR filter (Neewer) is mounted on a stand and controlled using DigiCamControl software. The experiment is performed in a dark room that is free of visible light.

This technique is mainly applied to unaltered texts to determine whether there are similarities between the ink strokes of the different pens under 850 nm IR radiation, based on whether they absorb or reflect this wavelength. Some inks may be visible or invisible during IR photography.

#### IR digital photography in luminescence mode

A full spectrum camera and 720 nm highpass IR filter are used in a dark room with incident light of 505 nm (i.e. cyan) shining on the samples [10], and the strokes that emit IR fluorescence are recorded. This technique can help verify the possibility visualizing the written content in a colourless state, specifically if they present IR luminescence after the absorption of visible light. We also consider the differences in fluorescence intensity, although it is not measured directly. For this reason, we discriminate whether there is a truly notable and contrastable distinction, and this is why the entire sample (i.e. all ink strokes from each pen) are captured in the same photography under the same lighting conditions.

## Results

### Unaltered text

#### Naked eye observation

The thermochromic inks in the unaltered state do not present characteristics that can be discerned by the naked eye.

#### Observation using a stereoscopic magnifying glass

The different characteristics observed in the writing samples are described separately below to help clarify what our observations refer to.

#### Pasty appearance

**Figure 4:**
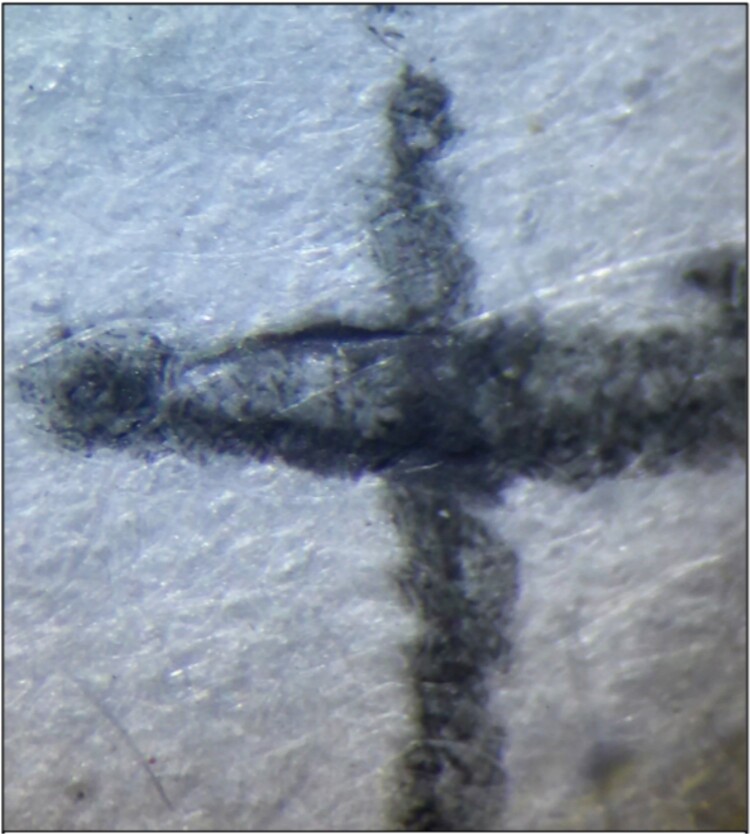
Unaltered strokes produced using the Pilot FriXion pen, showing the pasty appearance and relief in the areas of ink accumulation. Photography: LG-D722AR camera; exposure time: 1/29 s; F: 2.6; ISO: 100.

**Figure 5:**
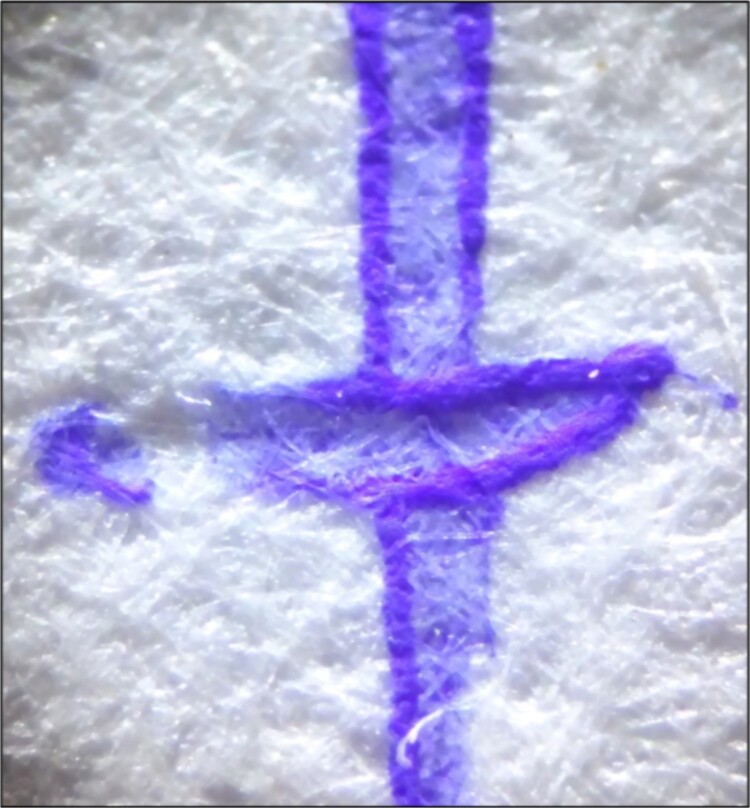
Unaltered strokes produced using the Faber Castell Magic pen, showing the pasty appearance and less ink accumulation in the internal zone of the strokes. Photography: LG-D722AR camera; exposure time: 1/16 s; F: 2.6; ISO: 300.

**Figure 6:**
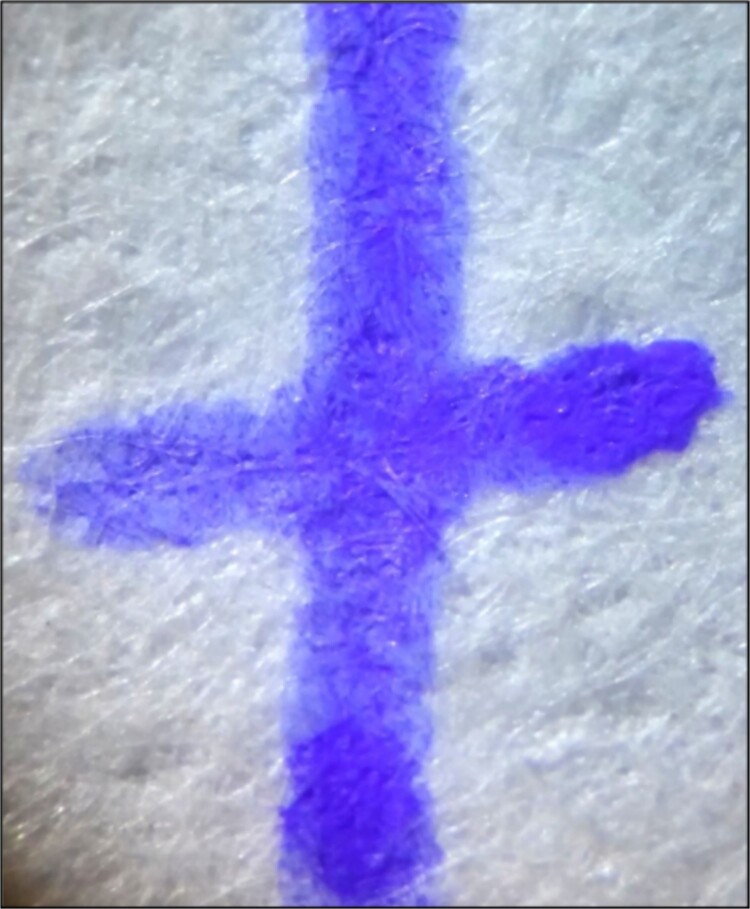
Unaltered strokes produced using the Simball Genio 2G Teen 0.7 pen, showing ink accumulation at the end of the stroke path. Photography: LG-D722AR camera; exposure time: 1/16 s; F: 2.6; ISO: 300.

This terminology was originally used to describe the strokes made using the Pilot FriXion pen, exhibiting an appearance like the surface of a “caoutchouc”, which is a pasty surface [4]. To recognize this sign, we must pay attention to locations where the greatest accumulation of ink is found, such as the external zones of the strokes (Figures 4 and 5) or the ends of the stroke paths (Figure 6).

#### Internal zone of the stroke with less ink accumulation

The reduced accumulation of ink in the internal zone of the pen strokes has been previously described, as mentioned in the literature review [4]. We also consider the frequency of this characteristic in the written strokes for the different brands and models. However, a devoted study is required to analyse this individual characteristic because it was necessary to conduct all the strokes with a similar speed and pressure. From our results, the Filgo Borrax SE 0.7 and Faber Castell Magic pens (Figure 5) presented this peculiarity in all their strokes.

#### Ink accumulation at the end of the stroke path

Figure 6 showed that the end of the stroke path has a greater presence of ink accumulation. This feature also allows the observer to determine the direction of the stroke production.

#### Relief in the areas of ink accumulation

In several cases, in both the external zone of the stroke and at the end of the stroke path, a slight relief can be observed using optical instruments and raking light (Figures 4–6). Once again, a specific study is required regarding this characteristic because variations in the writing speed and pressure may significantly affect the results. Nonetheless, the distinction of this characteristic in the writing samples is important evidence when it comes to recognizing a thermochromic ink stroke, even though the absence of this characteristic does not definitively indicate that a specific stroke was not produced by this type of pen. Overall, the Trabi Ghost pen (black) presented this characteristic most noticeably and in all areas of ink accumulation.

Table 1 provides a summary of the observations; the presence of the characteristics described above is marked with a cross according to the pen used.

**Table 1:** Characteristics of the strokes observed using a stereoscopic magnifying glass.

Brands	Pasty appearance	Internal zone of some strokes has less ink accumulation	Internal zone of all strokes has less ink accumulation	Ink accumulation at the end of the stroke path	Relief in some areas of ink accumulation	Relief in all areas of ink accumulation
Black colour						
Pilot FriXion	×	×		×	×	
Trabi Ghost	×			×		×
Keyroad Erasable Gel Pen	×			×	×	
Simball Genio 2G 0.7	×	×		×	×	
Filgo Borrax RT Ret.	×	×		×	×	
Zuixua Animal School K 1300 0.5 mm	×	×		×		
Blue colour						
Trabi Ghost	×	×		×	×	
Simball Genio 2G	×	×		×	×	
Simball Genio T 2G	×	×		×	×	
Filgo Borrax RT Ret.	×	×		×	×	
Filgo Borrax SE	×		×	×	×	
A+ Plus Erasable (tube: light blue)	×	×		×	×	
A+ Plus Erasable (tube: pink)	×			×		
Faber Castell Magic	×		×	×	×	
Sabonis Office Line	×	×		×		
Percentage	100%	67%	13%	100%	73%	7%

#### UV radiation at 365 and 254 nm

The examined inks do not emit visible fluorescence through this stimulus, and thus we cannot distinguish any striking characteristics.

#### Digital IR photography in reflection mode

**Figure 7:**
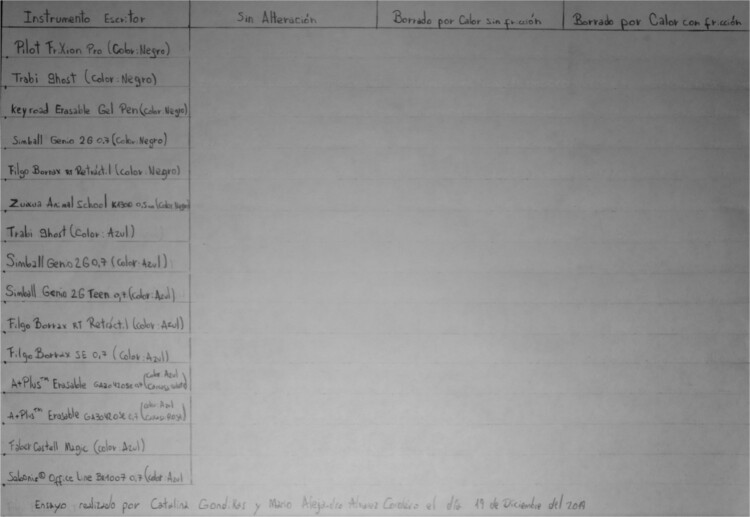
Table A, unaltered strokes (second column). Photography: LG-D722AR camera; exposure time: 1/16 s; F: 2.6; ISO: 300.

This technique is used to check how the strokes react under 850 nm IR radiation. In all cases, neither the unaltered inks nor the reappeared inks were visible in the photographic record (Figure 7). This is an important general characteristic for unaltered strokes.

#### IR digital photography in luminescence mode

As a general characteristic of the unaltered sampling, all inks presented IR luminescence under incident radiation of 505 nm cyan light. Because we do not have the necessary instruments to measure the luminescence intensity, we cannot quantify the results. However, similar results are grouped and differentiated based on clearly noticeable distinctions (Table 2).

**Table 2:** Incident radiation of 505 nm cyan light, capturing IR luminescence using a full spectrum camera and 720 nm high-pass IR filter.

Thermochromic ink pen	Luminescence intensity
Black colour	
Pilot FriXion	B
Trabi Ghost	A
Keyroad Erasable Gel Pen	D
Simball Genio 2G 0.7	D
Filgo Borrax RT Ret.	A
Zuixua Animal School K 1300 0.5 mm	D
Blue colour	
Trabi Ghost	C
Simball Genio 2G	C
Simball Genio T 2G	C
Filgo Borrax RT Ret.	C
Filgo Borrax SE	C
A+ Plus Erasable (tube: light blue)	C
A+ Plus Erasable (tube: pink)	C
Faber Castell Magic	C
Sabonis Office Line	C

Reference A: higher than B, C, and D; Reference B: higher than C and D; Reference C: higher than D; Reference D: lowest.

**Figure 8:**
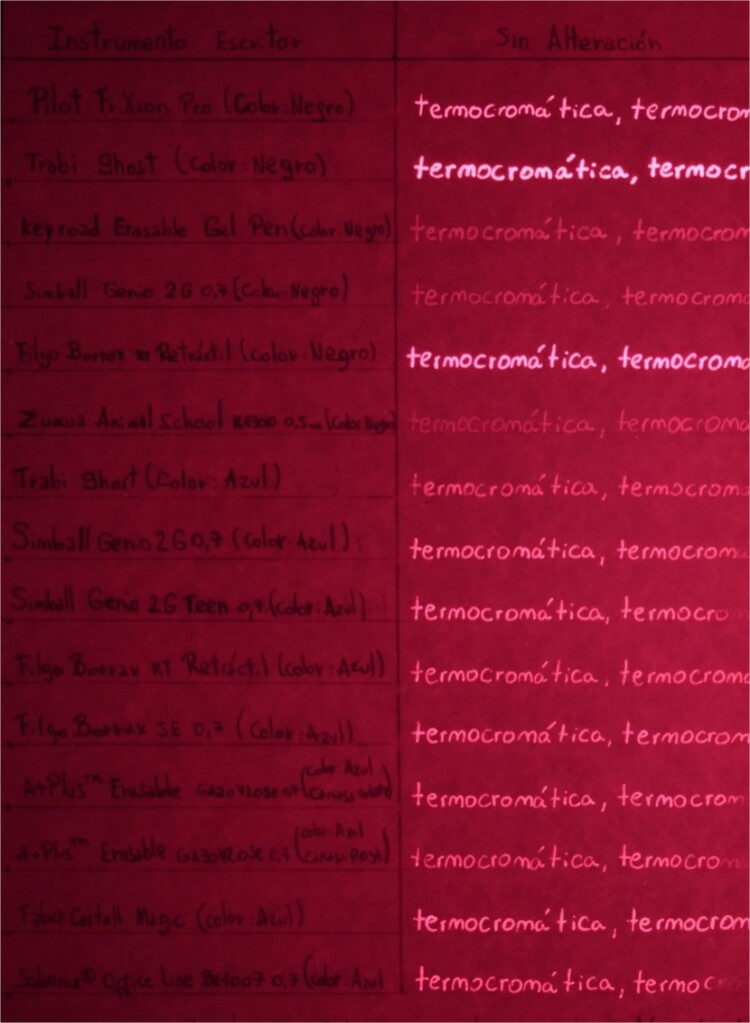
Table A, photograph in a dark room with incident cyan light (505 nm). Photography: Nikon D3400 full spectrum camera and 720 high-pass IR filter.

Table 2 shows that the blue inks have greater homogeneity than the black inks in terms of the luminescence intensity. Although this is an important general characteristic of blue thermochromic ink pen, it cannot be used to determine the specific model. In contrast, the black inks exhibit greater variations in intensity, with a lower intensity for the strokes made using the Keyroad Erasable Gel Pen; Simball Genio 2G 0.7, and Zuixua Animal School K 1300 0.5 mm pens, and the greatest intensity for the strokes made using the Trabi Ghost and Filgo Borrax RT Retractable pens (Figure 8).

### Colourless text

#### Naked eye observation

**Figure 9:**
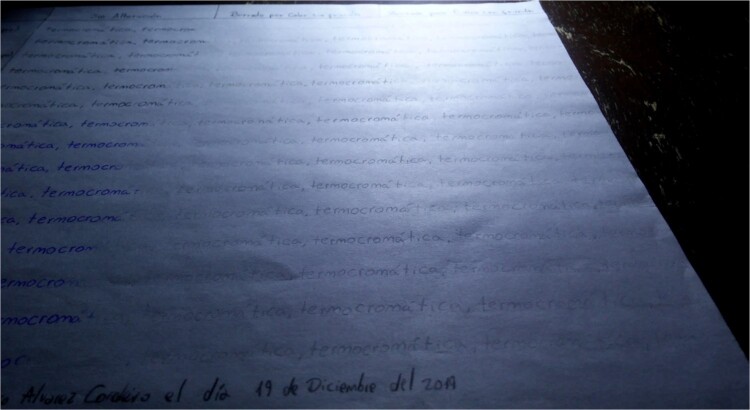
Table A, photography in a dark room using oblique white light opposite to the angle of observation. Photography: LG-D722 camera; exposure time: 1/29 s; F: 2.6; ISO: 100.

By naked eye observation, the colourless texts written using the Filgo Borrax RT Retractable (black colour) and Trabi Ghost (blue colour) pens exhibit visible residues (Figure 2). However, through oblique lighting, all of the writing can be made visible. This phenomenon is perceived as long as the incident light beam is positioned at the opposite angle to our observation (Figure 9).

Notably, this observation alone is not sufficient to determine the type of writing instrument that was used. These inks are analysed on white paper without any type of background, but when we have a paper with colour backgrounds or offset screening the contrast of the ink is reduced. These inks become imperceptible to the naked eye.

Another way to detect the text is by placing the incident light at a low angle because people can see the physical indentations produced during writing. Moreover, the visibility of the ink could be recovered at −18°C in all cases, regardless of the heating method (Table 3).

**Table 3:** Naked eye observation of colourless text (regardless of heating method).

Thermochromic ink pen	Visible under incident white light	Visible with oblique white light opposite to the angle of observation
Black colour		
Pilot FriXion		×
Trabi Ghost		×
Keyroad Erasable Gel Pen		×
Simball Genio 2G 0.7		×
Filgo Borrax RT Ret.	×	×
Zuixua Animal School K 1300 0.5 mm		×
Blue colour		
Trabi Ghost	×	×
Simball Genio 2G		×
Simball Genio T 2G		×
Filgo Borrax RT Ret.		×
Filgo Borrax SE		×
A+ Plus Erasable (tube: light blue)		×
A+ Plus Erasable (tube: pink)		×
Faber Castell Magic		×
Sabonis Office Line		×

#### Using a stereoscopic magnifying glass

##### *Indented impressions (raking light)*

The presence of indentations depends on the writing pressure and writing surface. For this test, the use of a raking light was necessary to observe the indentations.

##### *Slight relief in areas of ink accumulation (Figures 10–12)*

**Figure 10:**
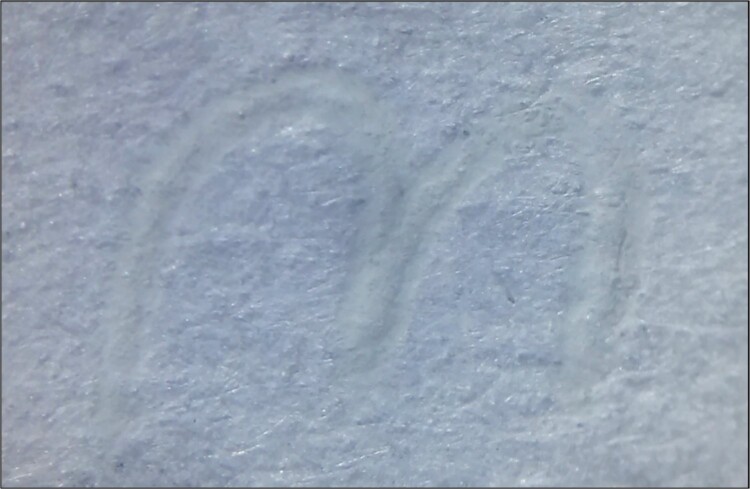
Colourless strokes produced without friction. Thermochromic ink pen: Faber Castell Magic (blue colour); photomicrography (20×): LG-D722AR camera; exposure time: 1/40 s; F: 2.6; ISO: 100.

**Figure 11:**
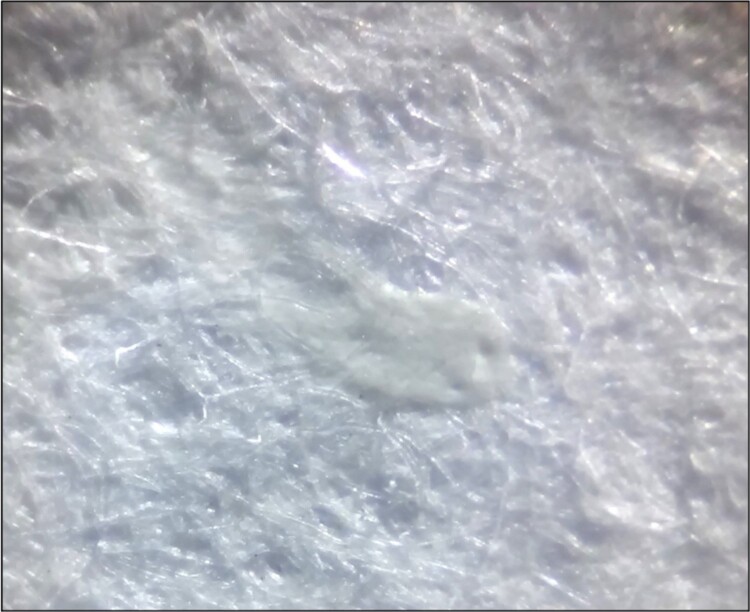
Colourless terminal stroke produced by friction. Thermochromic ink pen: Sabonis® Office Line BR1007 0.7; photomicrography (20×): LG-D722AR camera; exposure time: 1/24 s; F: 2.6; ISO: 200.

**Figure 12:**
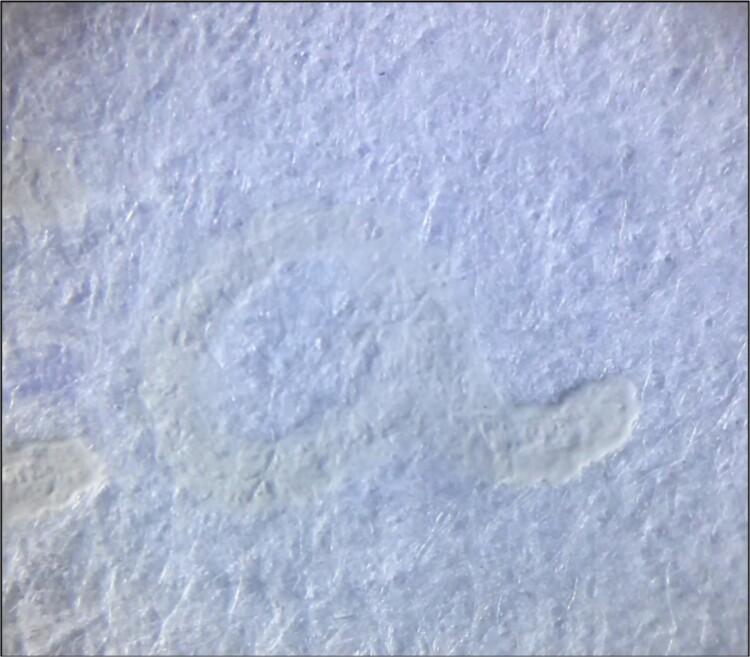
Colourless strokes produced without friction. Thermochromic ink pen: Trabi Ghost (black colour); photomicrography (20×): LG-D722AR camera; exposure time: 1/60 s; F: 2.6; ISO: 100.

The frequency of this feature throughout each of the texts varies depending on the model of the writing tool (Table 4). However, slight relief is noticeable at the ends of some stroke paths when observed under raking light.

**Table 4:** Slight relief in areas of ink accumulation.

Thermochromic ink pen	Colourless strokes produced by friction	Colourless strokes produced without friction
Black colour		
Pilot FriXion	×	×
Trabi Ghost	×	×
Keyroad Erasable Gel Pen	×	×
Simball Genio 2G 0.7	×	×
Filgo Borrax RT Ret.	×	×
Zuixua Animal School K 1300 0.5 mm		
Blue colour		
Trabi Ghost	×	×
Simball Genio 2G	×	
Simball Genio T 2G	×	×
Filgo Borrax RT Ret.	×	×
Filgo Borrax SE	×	×
A+ Plus Erasable (tube: light blue)	×	
A+ Plus Erasable (tube: pink)		
Faber Castell Magic	×	×
Sabonis Office Line		
Total	80%	67%

In the case of the Filgo Borrax SE 0.7 and Faber Castell Magic pens, this feature is most obvious in the external zone of the stroke. It becomes more difficult to visualize this characteristic in texts manipulated by friction. The only pens with strokes where this feature was not perceived in any of the manipulation modalities are the Zuixua Animal School K1300 0.5 mm (black colour) and A+ Plus Erasable GA304205E 0.7 (blue colour, pink tube) pens.

##### *Slight yellow tonality*

A slight yellow tone is observed in five cases (Table 5) but is particularly striking in the black Trabi Ghost and Filgo Borrax RT Retractable pens, regardless of the two manipulation modalities (Figure 12). This yellow colouration is less noticeable for the Pilot FriXion (black), Sabonis Office Line BR1007 0.7 (blue), and Filgo Borrax RT Retractable (blue) pens.

**Table 5:** Slight yellow tonality.

Thermochromic ink pen	Colourless strokes produced by friction	Colourless strokes produced without friction
Black colour		
Pilot FriXion	×	×
Trabi Ghost	×	×
Keyroad Erasable Gel Pen		
Simball Genio 2G 0.7		
Filgo Borrax RT Ret.	×	×
Zuixua Animal School K 1300 0.5 mm		
Blue colour		
Trabi Ghost		
Simball Genio 2G		
Simball Genio T 2G		
Filgo Borrax RT Ret.	×	×
Filgo Borrax SE		
A+ Plus Erasable (tube: light blue)		
A+ Plus Erasable (tube: pink)		
Faber Castell Magic		
Sabonis Office Line	×	×
Total	33%	33%

##### *Slight light blue tonality*

A light blue tone is observed in the texts written using the A+ Plus Erasable GA304205E 0.7 pens (blue colour, both light blue and pink tubes), erased under heat without friction (Table 6). Additionally, according to previous work, “the greater the interval between the production of the writing and its erasure, the more visible signs are observed” [3]. Overall, these signs are important to identify the presence of text in a colourless state, especially for subsequent confirmation with other applied techniques.

**Table 6:** Slight light blue tonality.

Thermochromic ink pen	Colourless strokes produced by friction	Colourless strokes produced without friction
Black colour		
Pilot FriXion		
Trabi Ghost		
Keyroad Erasable Gel Pen		
Simball Genio 2G 0.7		
Filgo Borrax RT Ret.		
Zuixua Animal School K 1300 0.5 mm		
Blue colour		
Trabi Ghost		
Simball Genio 2G		
Simball Genio T 2G		
Filgo Borrax RT Ret.		
Filgo Borrax SE		
A+ Plus Erasable (tube: light blue)	×	
A+ Plus Erasable (tube: pink)	×	
Faber Castell Magic		
Office Line		
Total	13%	0%

#### UV radiation at 365 and 254 nm

**Figure 13:**
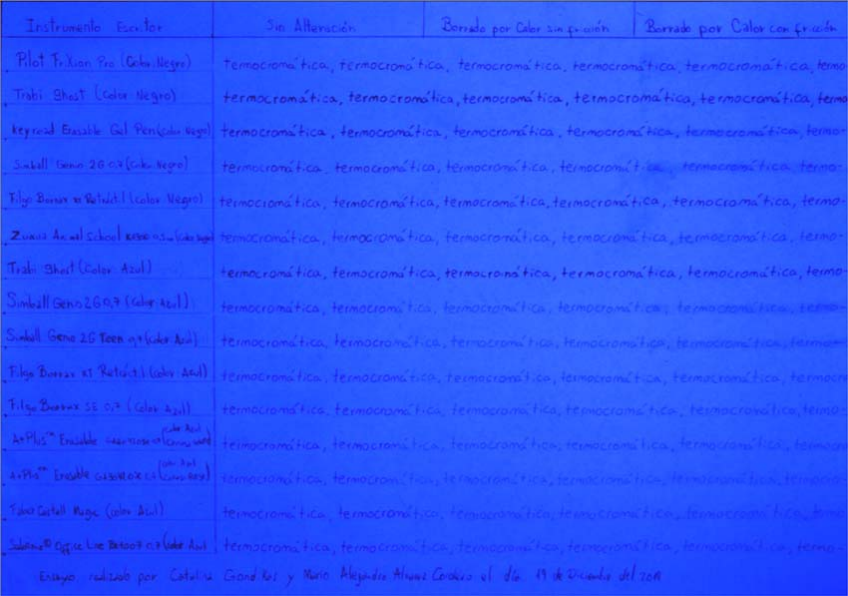
Table A, photography captured in a dark room under incident UV light (365 nm). Photography: LG-D722AR camera; exposure time: 8 s; F:9; ISO: 100.

In all cases, the colourless text could be visualized using UV radiation. The ink appears opaque, contrasting with the blue fluorescence of the paper (Figure 13). Backgrounds that are not fluorescent under UV excitation would make it impossible to detect this phenomenon.

**Figure 14:**
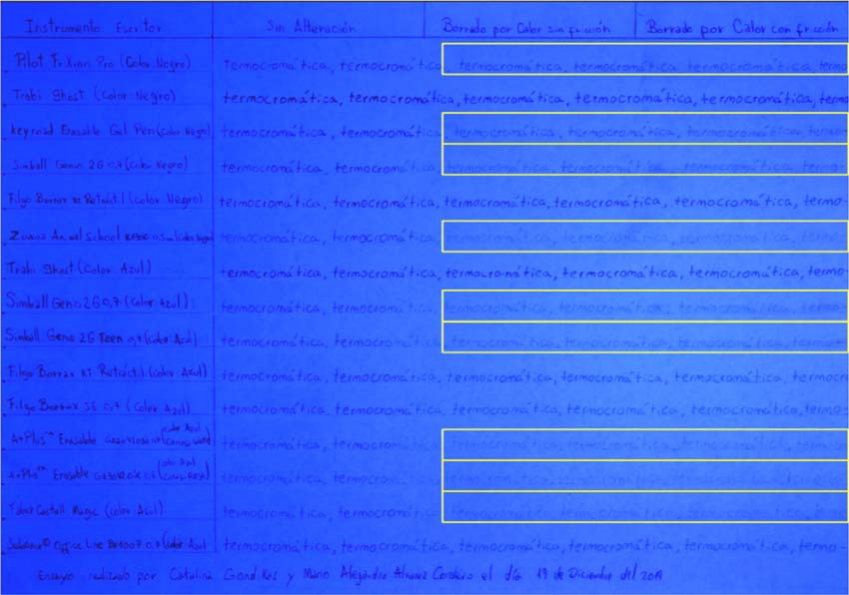
Table A, photography captured in a dark room under incident UV light (254 nm). Photography: LG-D722AR camera; exposure time: 3 s; F: 9; ISO: 100.

With the change in radiation from 365 to 254 nm light, the pens reduce their chromatic contrast with the fluorescence of the paper, especially the Pilot FriXion (black colour), Keyroad Erasable Gel Pen (black colour), Simball Genio 2G 0.7 (black colour), Zuixua Animal School K 1300 0.5 mm (black colour), Simball Genio 2G 0.7 (blue colour), and Simball Genio Teen 2G 0.7 (blue colour) pens (Figure 14).

#### IR digital photography in reflection mode

The text remains invisible using this technique (Figure 7, third and fourth column).

#### IR digital photography in luminescence mode

**Figure 15:**
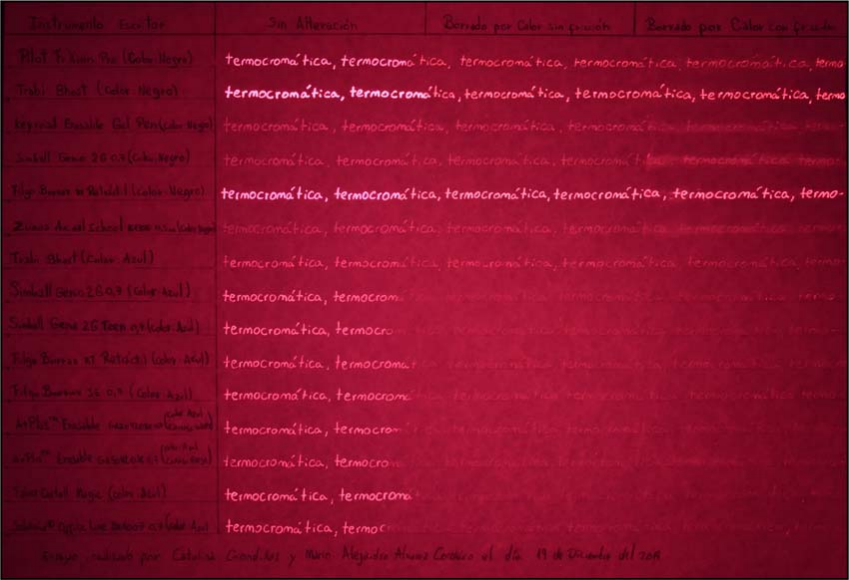
Table A, photography in a dark room using incident light of 505 nm. Photography: NIKON D3400 full spectrum camera and high-pass IR filter (720 nm); exposure time: 15 s; F: 9; ISO: 100.

Inks in the colourless state present IR luminescence under the irradiation of 505 nm cyan light, and the luminescence intensities are compared between the different inks in Table 7. This comparison may aid in the identification of the writing tool, find both the erased and unaltered text. All the blue inks in the sample present a slight fluorescence intensity under illumination at 505 nm, except Trabi Ghost. This is why it is essential to use long exposure times, so that the fluorescence is noticeable (Figure 15). This phenomenon may not be observable in “live view”, in some cases.

**Table 7:** Incidence of 505 nm cyan light, capturing infrared radiation (IR) luminescence using a full spectrum camera and 720 nm high-pass IR filter.

Thermochromic ink pen	Luminescence intensity
Black colour	
Pilot FriXion	B
Trabi Ghost	A
Erasable Gel Pen	C
Simball Genio 2G 0.7	C
Filgo Borrax RT Ret.	A
Zuixua Animal School K 1300 0.5 mm	C
Blue colour	
Trabi Ghost	C
Simball Genio 2G	D
Simball Genio T 2G	D
Filgo Borrax RT Ret.	D
Filgo Borrax SE	D
A+ Plus Erasable (tube: light blue)	D
A+ Plus Erasable (tube: pink)	D
Faber Castell Magic	D
Sabonis Office Line	D

Reference A: higher than B, C, and D; Reference B: higher than C and D; Reference C: higher than D; Reference D: lowest.

**Figure 16:**
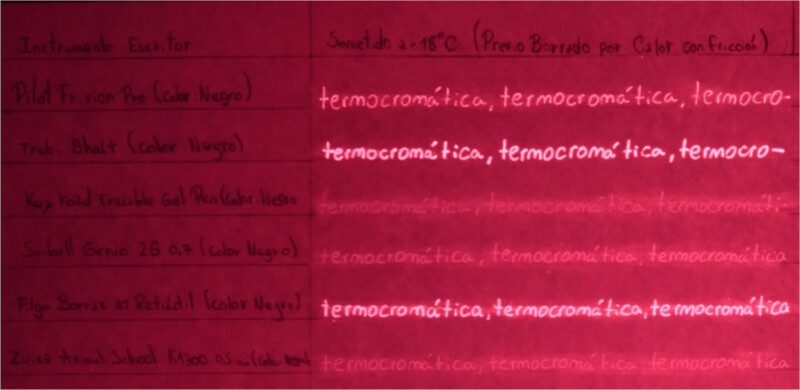
Table B, photography in a dark room using incident light of 505 nm. Photography: Nikon D3400 full spectrum camera and high-pass IR filter (720 nm); exposure time: 15 s; F: 9; ISO: 100.

Finally, we highlight a finding that may be used to distinguish the type of erasure. Specifically, after the colour has reappeared, blurring of the luminescence is observed for the text erased using friction, particularly for the black Pilot FriXion, Keyroad Erasable Gel Pen, Simball Genio 2G 0.7, and Zuixua Animal School K 1300 0.5 mm pens (Figure 16).

## Conclusions

Although the sample size was restricted to 15 writing tools in this study, this is the largest sampling used to date. The most important characteristics present in strokes produced using the thermochromic ink pens, both mentioned in the literature review and observed herein, are summarized as follows:

### General characteristics

#### Unaltered text

When observed with a stereoscopic magnifying glass, inks present a “pasty” appearance, as previously described in the literature. In addition, the accumulation of ink on edges is a common characteristic (Table 8). In the latter case, it is necessary to evaluate the influence of writing pressure. The ink is not visible in the photographic record using IR digital photography in reflection mode (high-pass IR filter, 720 nm, and incident radiation of 850 nm), but the IR luminescence of the ink can be captured under 505 nm cyan light using a full spectrum camera with a 720 nm highpass IR filter.

**Table 8:** General characteristics of the strokes produced using thermochromic ink pens.

Characteristics	Condition
Unaltered	Pasty appearance Ink accumulation at the end of the stroke path Under 860 nm, captured by Infrared photography, becomes invisible Under 505 nm, captured by IR photography, presents luminescence
Colourless (heat and friction/heat without friction)	Visible using oblique white light opposite to the angle of observation Visible under UV light (365 and 254 nm) Visible due to the luminescence emitted by the ink under 505 nm, reaction captured by IR photography
Reappeared	−18°C

#### Text in a colourless state, manipulated by heat with and without friction

When the strokes are located on a non-printed area on white paper, they can be visualized based on the contrast difference using oblique light placed at an angle opposite to that of the observation. This oblique light must also be the only light source. Under UV radiation of 254 and 365 nm, the strokes in a colourless state can be visualized in contrast with the blue fluorescence of the paper, this will be not useful on UV-dull paper. The ink in a colourless state presents IR luminescence when subjected to 505 nm cyan light, recorded using a full spectrum camera with a high-pass IR filter of 720 nm. However, there are cases where the intensity is so low that it cannot be observed in “live view”, and therefore, the exposure time must be sufficiently long. All of the aforementioned characteristics are relevant in determining the general existence of strokes produced by thermochromic ink pens because they were witnessed in 100% of the cases.

#### Reappeared text

The ink in a colourless state can become visible again with exposure to temperatures of −18°C; however, some inks present less intensity compared with the unaltered state. This indicates that the examiner should avoid using incandescent lights because the presence of heat may alter the evaluation.

### Specific characteristics

These characteristics are not observed in all cases but are important because of their unique occurrence in the set of pens studied.

#### Unaltered text

A slight relief in areas of ink accumulation is observed in 80% of the cases, with variation found in the frequency of appearance within the analysed text.

Less ink accumulation is observed in the internal zone of the pen stroke in 80% of the cases; however, only 13% presented this feature in all the strokes made. It is necessary to vary the writing speed and pressure to determine whether this is a constant feature.

#### Text in a colourless state, manipulated by heat with and without friction

The presence of indentations and residues must be considered, but this is also dependent on the writing pressure of the writer and the elapsed time between writing and erasing [3]. In 60% of the cases, a decrease in contrast is observed between the stroke and the blue fluorescence emitted by the paper during the change in UV radiation from 365 to 254 nm.

Blue inks, in a colourless state, have a relatively low IR luminescence intensity. Black inks present more variations in intensity among them.

#### Text in a colourless state, manipulated by heat with friction

For black inks, friction can result in the appearance of “blurry” IR luminescence in the reappeared text, indicating the action exerted by the eraser of the thermochromic pen.

Overall, three techniques are presented to visualize the manipulated text produced using thermochromic ink: oblique white illumination opposite to the angle of observation, UV radiation of 365 and 254 nm, and IR photography in luminescence mode. Concluding the non-existence of thermochromic ink without covering all these techniques is an error that must be avoided, especially when considering paper with printed backgrounds or without optical brightening agents, in which cases the visual contrast between the ink and the background can be diminished.

## References

[1] Abd-ElAziz Abd-ElZaher M . Different types of inks having certain medico legal importance: deciphering the faded and physically erased hand writing. Egypt J Forensic Sci. 2014;4:39–44.

[2] Eldebss TMA , El-Zawawy WK, Gazy MB, et al. Using an erasable ink to forge documents, medico-legal study on evaluating them in detection and prevention the forgery. J Am Sci. 2015;11:30–46.

[3] Escobedo G . Borrado y Revenido de Tinta gel Termosensible. Revista Skopein. 2017;XVI:6–13. Available from: http://www.skopein.org. Spanish.

[4] Ezcurra Gondra M , Grávalos GR. Instrumentos de Escritura Manual y Sus Tintas. Argentina: Editorial La Rocca, 2010. Spanish.

[5] PILOT Corporation . Secretos de la Tinta, Available from: http://www.frixion.jp/sp/ink/. Spanish.

[6] Masuda M . The Science Behind FriXion Erasable Pens, 2016, Available from: https://www.nippon.com/en/features/c00520/

[7] Chayal VM , Handa DR, Singh J, et al. A sensitive non-destructive method for detection of document frauds using thermal ink. Aust J Forensic Sci. 2016;48:601–612.

[8] Teo CH , Mohamad Noor SNM, Wong KY. Ink that disappears: examination of questioned documents related to Frixion ink in Malaysia. Can Soc Forensic Sci J. 2017;50:146–155.

[9] Das A . Effect of thermochromic ink on different types of paper. Indian J Forensic Med Pathol. 2021;14:403–407.

[10] Narcotti G . La fotografía Pericial. Argentina: Editorial Dos y una Ediciones Argentinas, 2013. Spanish.

